# Bled dry? An audit of blood sampling practices on an adult intensive therapy unit

**DOI:** 10.1186/cc13276

**Published:** 2014-03-17

**Authors:** RK Vincent, P Temblett

**Affiliations:** 1 ABMU Health Board, Swansea, UK

## Introduction

The aim of this audit was to evaluate whether guidelines produced in a local intensive therapy unit (ITU) with regard to blood sampling practices were being adhered to. The volume of blood taken and cost was also evaluated.

## Methods

A retrospective audit investigating the number of routine blood tests ordered on an ITU in January 2013 was performed. There were no exclusion criteria. Computer-based data collection systems were used to gather data with regard to patient details and when blood tests were processed. The collection bottles used were examined to see how much blood was needed to fill them. Thirty nurses were asked how much 'dead space' blood they discarded, and an average was recorded. The cost of the blood tests was also calculated. Following a period of education regarding the contents of the guidelines, this was re-audited in July 2013 retrospectively.

## Results

The initial audit examined 901 patient-days. Urea and electrolytes (U&Es) and full blood counts (FBC) were requested in line with the guidelines. Liver function tests (LFTs), bone profile, magnesium and a clotting screen were ordered approximately four times more than advocated. It was shown that many bone profiles and magnesium tests were probably inappropriate requests. Moreover, twice as much blood was taken from patients compared with that recommended by guidelines (almost 16 litres in total in January). The cost of the routine blood tests in January was €11,019. If guidelines had been followed, the estimated yearly saving would be €65,588. During the repeat audit, 731 patient-days were examined. The amount of times a U&E or FBC were requested was largely unchanged, but the amount of times a LFT, bone profile, magnesium and clotting screen were ordered reduced by approximately 50%. Almost one-third more blood was taken from patients when compared with the suggested volume in the guidelines. The cost of the blood tests done in July was €5,423. Despite an improvement in the frequency of blood testing, an estimated €21,907 per year could still be saved.

## Conclusion

The results underline that the unit's guidelines were not being followed. The re-audit does show an improvement in adherence. Patients are being exposed to unnecessary blood tests, which not only is implicated in iatrogenic anaemia, but also places a significant financial burden on the department. Continued staff education and encouragement are required in order to aid the transition from current to recommended practice.

